# Comparisons of different indices of low muscle mass in relationship with cardiometabolic disorder

**DOI:** 10.1038/s41598-018-37347-3

**Published:** 2019-01-24

**Authors:** Ju Young Kim, Sohee Oh, Hwa Yeon Park, Ji Hye Jun, Hwa Jung Kim

**Affiliations:** 1 Department of Family Medicine, Seoul National University Bundang Hospital, Seoul National University College of Medicine, Seongnam, Korea; 2 Department of Biostatistics, Seoul Metropolitan Government Seoul National University Boramae Medical Center, Seoul, Korea; 3 Department of Family Medicine, Seoul National University Bundang Hospital, Seongnam, Korea; 4 Deparment of Family Medicine, Chamjoeun Hospital, Gwangju, Korea; 5 Department of Preventive Medicine, University of Ulsan College of Medicine, Seoul, Korea

**Keywords:** Metabolic syndrome, Risk factors

## Abstract

This study aimed to evaluate the most valid index among various indices of low muscle mass in assessing cardiometabolic risks in a Korean population. Appendicular lean mass index (ALMI, kg/m^2^), fat mass index (FMI, kg/m^2^), FMI-adjusted ALMI (ALM_fmi_), ratio of ALM to weight index (ALM_wt_), ratio of ALM to body mass index (ALM_bmi_) and ratio of ALM to truncal fat index (ALM_trunkfat_) were measured by dual energy X-ray absorptiometry in 17,870 participants from 2008 to 2011. We adopted all the aforementioned indices of low muscle mass expressed as sex- and age-specific standard deviation scores (Z-scores). Low muscle mass for age was defined as Z-score <−1. The prevalence of low muscle mass was approximately 16% across all indices. Low muscle mass defined by ALMI had low muscle mass and low fat mass, and ALM_fmi_ had low muscle mass at the same FMI. However, low muscle mass defined by ALM_wt_, ALM_bmi_ and ALM_trunkfat_ had similar muscle mass with high FMI. The receiver operating characteristic curve in metabolic syndrome showed that the ALM_trunkfat_ was 0.74 in male and 0.69 in female, indicating that ALM_trunkfat_ was the best discrimination index for metabolic syndrome. This study showed that ALM_trunkfat_ could be a useful indicator for screening cardiometabolic risk factors, particularly in normal or overweight Asian population.

## Introduction

The decrease in skeletal muscle has a complex multifactorial pathogenesis. The prevalence of low muscle mass increases in patients with cancer, cardiovascular disease and chronic obstructive airway disease^1,2^. It also carries poor prognosis in terms of frailty, hospitalisation and mortality in several settings^3^. Low muscle mass has recently been recognised as a disease and assigned the code M62.84 in the ICD-10 (the International Statistical Classification of Disease and Related Health Problems)^4^.

Currently, no general consensus exists on the definition of muscle mass depletion, partly due to diverse measurement tools in assessing skeletal muscle mass and conceptual confusion between the disease or condition related to low muscle mass and frailty^5^. Imaging technologies, such as computed tomography and magnetic resonance imaging, are precise and valid but cannot be used in general population because of high cost and lack of feasibility^6^. Therefore, most operational definitions of low muscle mass have been assessed by dual energy X-ray absorptiometry (DXA) or bioelectrical impedance analysis (BIA)^7^. However, DXA and BIA have limitations in assessing muscle mass. Fat-free mass assessed by DEXA or BIA is a heterogeneous compartment that consists of muscle and connective tissues. It does not correctly reflect muscle mass with increasing age, advancing body fat mass or disturbance of hydration in fat-free mass such as chronic heart and renal failure^8^.

Another barrier in defining low muscle mass is the diversity of body composition parameters used and discrepancies in the method of normal range definition^8^. According to age, height, sex, ethnicity and body mass index (BMI), normal distribution of muscle mass with fatness is quite different, and universal reference criteria are not well defined^8,9^. These diversities lead to large variation up to 26-fold in the prevalence of sarcopenic obesity and no predictive roles in self-reported physical difficulties and knee extension strength^10,11^. The final challenge in defining low muscle mass is that it presents as a predictor of various phenotypic outcomes, such as cardiometabolic risks^12,13^, frailty^14^ or cognitive dysfunction^15^ usually in older adults and survival or functional declines in cancer patients^16^. In particular, cardiometabolic risk factors related to low muscle mass show stronger associations in younger adults than in older adults^17^. However, few studies have focused on the outcome of function or cardiovascular risk assessment in younger adults.

Whether assessing the phenotypes of low muscle beyond BMI and waist circumference can add another insight into cardiometabolic risk assessment requires evidence, but some studies suggest that low muscle with high fat is more closely associated with insulin resistance than with obesity^12,18,19^. Thus, in this study, we aimed to focus on evaluating the most valid index among various indices of low muscle mass in assessing cardiometabolic risks in a Korean population.

## Results

The characteristics of the participants of the Korea National Health and Nutrition Examination Surveys (KNHANES) are summarised in Table 1. The prevalence of metabolic syndrome among KNHANES population was 22% in male and 19% in female.

**Table 1:** Anthropometric, clinical and behavioural characteristics of 17,870 participants of the Korean National Health and Nutrition Examination Survey 2008–2011.

Variables	Male	Female
Mean (SE) or frequency (%)	n = 7667	n = 10203
Age, year	44.34 (0.28)	46.61 (0.27)
Body height (cm)	170.62 (0.10)	157.04 (0.10)
Body weight (kg)	69.81 (0.15)	56.89 (0.11)
Waist circumference (cm)	83.86 (0.14)	77.68 (0.16)
BMI (kg/m^2^)	23.94 (0.04)	23.09 (0.05)
ALM (kg)	22.55 (0.06)	14.43 (0.03)
ALMI (kg/m^2^)	7.72 (0.02)	5.85 (0.01)
FM (kg)	14.40 (0.09)	17.90 (0.08)
FMI (kg/m2)	4.94 (0.03)	7.28 (0.04)
ALM/weight	0.32 (0.00)	0.26 (0.00)
ALM/BMI (m^2^)	0.95 (0.00)	0.63 (0.00)
ALM/truncal fat	3.20 (0.03)	1.75 (0.01)
Clinical factor		
Fasting blood glucose (mg/dL)	98.26 (0.32)	94.97 (0.24)
HOMA-IR	2.44 (0.02)	2.39 (0.03)
HDL cholesterol (mg/dL)	49.69 (0.19)	55.38 (0.17)
Triglyceride (mg/dL)	158.21 (2.00)	111.09 (1.03)
Systolic blood pressure (mmHg)	117.68 (0.28)	114.25 (0.30)
Diastolic blood pressure (mmHg)	77.28 (0.21)	72.67 (0.17)
Metabolic syndrome (%)	1922 (21.9)	2272 (19.1)
Behavioural factor		
Current smoker (%)	4745 (63.4)	787 (8.7)
Drinking problem (%)	1585 (26.1)	415 (7.6)
No regular physical activity (%)	3412 (44.3)	5139 (50.2)

BMI = body mass index; ALM = appendicular lean mass; ALMI = appendicular lean mass index; FM = fat mass; FMI = fat mass index; HOMA-IR = homeostatic model assessment for insulin resistance.

### Comparison of body composition between low muscle mass for age and control groups among different indices

Table 2 shows the characteristics between decreased muscle mass and normal muscle mass groups according to the five different definitions of muscle mass depletion. Since we adopted the definition of relative low muscle mass (Z score less than −1 SD) across age and sex, the prevalence of low muscle mass was approximately 16% among different indices. The difference in the average BMI was the highest in appendicular lean mass index (ALMI) criteria (20.5 in the low muscle mass group vs 24.2 in the control group) and the lowest in ratio of appendicular lean mass to body mass index (ALM_bmi_) criteria (25.5 in the low muscle mass group vs 23.2 in the control group).

**Table 2:** Comparison between participants with decreased muscle mass and control according to the different indices of low muscle mass.

Indices of muscle mass depletion	Variables	Male			Female		
		Low muscle mass as Z score ≤ −1SD	Control	p value	Low muscle mass as Z score ≤ −1SD	Control	p value
ALMI	N (%)	1176 (15.3)	6491 (84.7)		1568 (15.4)	8635 (84.6)	
	BMI	20.49 (2.14)	24.51 (2.78)	<0.0001	20.47 (2.32)	23.79 (3.30)	<0.0001
	ALMI	6.38 (0.43)	7.87 (0.76)	<0.0001	4.88 (0.28)	6.05 (0.62)	<0.0001
	FMI	4.13 (1.62)	5.12 (1.74)	<0.0001	6.58 (1.85)	7.53 (2.20)	<0.0001
	ALM_wt_	0.31 (0.03)	0.32 (0.03)	<0.0001	0.24 (0.02)	0.26 (0.03)	<0.0001
	ALM_bmi_	0.90 (0.13)	0.93 (0.11)	<0.0001	0.59 (0.09)	0.63 (0.09)	<0.0001
	ALM_trunkfat_	3.37 (1.87)	3.06 (1.53)	<0.0001	1.64 (0.69)	1.72 (0.73)	0.0023
ALMI_fmi_	N (%)	1382 (18.0)	6285 (82.0)		2157 (21.1)	8046 (78.9)	
	BMI	21.62 (2.60)	24.39 (2.92)	<0.0001	21.64 (3.02)	23.72 (3.34)	<0.0001
	ALMI	6.49 (0.49)	7.90 (0.75)	<0.0001	5.03 (0.38)	6.09 (0.62)	<0.0001
	FMI	4.93 (1.74)	4.98 (1.76)	0.3924	7.36 (2.21)	7.38 (2.17)	0.7456
	ALM_wt_	0.30 (0.03)	0.33 (0.03)	<0.0001	0.24 (0.02)	0.26 (0.02)	<0.0001
	ALM_bmi_	0.86 (0.11)	0.94 (0.12)	<0.0001	0.57 (0.08)	0.64 (0.09)	<0.0001
	ALM_trunkfat_	2.71 (1.40)	3.19 (1.61)	<0.0001	1.47 (0.62)	1.77 (0.74)	<0.0001
ALM_wt_	N (%)	1203 (15.7)	6464 (84.3)		1554 (15.2)	8649 (84.8)	
	BMI	26.19 (3.10)	23.47 (2.85)	<0.0001	25.82 (3.82)	22.82 (3.09)	<0.0001
	ALMI	7.35 (0.92)	7.70 (0.88)	<0.0001	5.60 (0.79)	5.92 (0.69)	<0.0001
	FMI	7.23 (1.46)	4.55 (1.46)	<0.0001	9.90 (2.13)	6.93 (1.86)	<0.0001
	ALM_wt_	0.28 (0.01)	0.33 (0.02)	<0.0001	0.22 (0.01)	0.26 (0.02)	<0.0001
	ALM_bmi_	0.80 (0.09)	0.95 (0.11)	<0.0001	0.52 (0.06)	0.64 (0.08)	<0.0001
	ALM_trunkfat_	1.74 (0.31)	3.36 (1.59)	<0.0001	1.06 (0.23)	1.82 (0.72)	<0.0001
ALM_bmi_	N (%)	1189 (15.5)	6478 (84.5)		1562 (15.3)	8641 (84.7)	
	BMI	25.69 (3.16)	23.57 (2.92)	<0.0001	25.35 (3.76)	22.90 (3.17)	<0.0001
	ALMI	7.40 (0.92)	7.69 (0.88)	<0.0001	5.61 (0.78)	5.92 (0.70)	<0.0001
	FMI	6.68 (1.72)	4.65 (1.57)	<0.0001	9.41 (2.25)	7.01 (1.96)	<0.0001
	ALM_wt_	0.29 (0.02)	0.33 (0.03)	<0.0001	0.22 (0.02)	0.26 (0.02)	<0.0001
	ALM_bmi_	0.77 (0.07)	0.95 (0.11)	<0.0001	0.51 (0.05)	0.64 (0.08)	<0.0001
	ALM_trunkfat_	1.97 (0.61)	3.32 (1.62)	<0.0001	1.15 (0.32)	1.81 (0.73)	<0.0001
ALM_trunkfat_	N (%)	1217 (15.9)	6450 (84.1)		1583 (15.5)	8620 (84.5)	
	BMI	26.74 (2.85)	23.36 (2.78)	<0.0001	26.63 (3.52)	22.66 (2.98)	<0.0001
	ALMI	7.61 (0.92)	7.65 (0.89)	0.0908	5.89 (0.84)	5.87 (0.70)	0.3071
	FMI	7.54 (1.23)	4.48 (1.37)	<0.0001	10.30 (1.87)	6.84 (1.77)	<0.0001
	ALM_wt_	0.28 (0.02)	0.33 (0.02)	<0.0001	0.22 (0.02)	0.26 (0.02)	<0.0001
	ALM_bmi_	0.82 (0.09)	0.95 (0.11)	<0.0001	0.54 (0.07)	0.64 (0.08)	<0.0001
	ALM_trunkfat_	1.67 (0.22)	3.38 (1.59)	<0.0001	1.02 (0.17)	1.83 (0.72)	<0.0001

BMI = body mass index; ALM = appendicular lean mass; ALMI = appendicular lean mass index; FM = fat mass; FMI = fat mass index; ALM_wt_ = appendicular lean mass to weight; ALM_bmi_ = appendicular lean mass to body mass index; ALM_trunkfat_ = appendicular lean mass to truncal fat.

When low muscle mass was classified by ratio of appendicular lean mass index adjusted for fat mass index (ALMI_fmi_), the ALMI was decreased in the low muscle mass group (the difference was −1.50 in male, −1.06 in female) given the same FMI between the groups. If the low muscle mass was classified by ratio of appendicular lean mass to weight (ALM_wt_) or ALM_bmi_ criteria, the difference in ALMI was −0.30 in male and −0.33 in female, whereas the difference in FMI was more prominent (2.03 to 2.68 in male, 2.40 to 2.97 in female). The biggest difference was noted when low muscle mass was classified by ratio of ALM to truncal fat index (ALM_trunkfat_) criteria; the difference in ALMI was −0.04 in male and 0.02 in female, whereas that in FMI was 3.06 in male and 3.46 in female.

### Association of different indices of low muscle mass with cardiometabolic risk factors

Each index of low muscle mass adjusted for age, sex and BMI was strongly associated with waist circumference or triglyceride (TG) in both male and female. However, the relationship with other cardiometabolic components was different among each index as shown in Table 3. The ALMI or ALMI_fmi_ was not associated with SBP and weakly associated with high-density lipoprotein (HDL) in male and weakly associated with HDL or TG in female. In general, the ALM_trunkfat_ was the only index strongly associated with each cardiometabolic component and inversely associated with homeostasis model assessment of insulin resistance (HOMA-IR) (β = −0.38 in male, β = −0.25 in female, p < 0.001) in both sexes.

**Table 3:** Age-, sex- and body mass index-adjusted linear and logistic models evaluating associations between several indices of low muscle mass with cardiometabolic components.

	Waist circumference	SBP	DBP	Glucose	HDL	TG	HOMA-IR	Metabolic syndrome by low muscle mass (Z < −1SD)
	β (95% CI)	β (95% CI)	β (95% CI)	β (95% CI)	β (95% CI)	β (95% CI)	β (95% CI)	OR (95% CI)
Male								
ALMI Z score (SD)	0.32*** (0.16, 0.47)	0.12 (−0.36, 0.60)	−063** (−0.97, −0.29)	−2.68*** (−3.39,−1.97)	0.60** (0.28, 0.92)	−12.66*** (−16.80, −8.52)	−0.14*** (−0.19, −0.09)	1.80*** (1.45, 2.25)
ALMI_fmi_ Z score	−0.42*** (−0.53, −0.30)	−0.14 (−0.49, 0.21)	−0.51*** (−0.75, −0.26)	–1.99*** (−2.51, −1.46)	0.74** (0.50, 0.97)	−11.07*** (−14.12, −8.04)	−0.16*** (−0.20,−0.12)	1.78*** (1.49, 2.12)
ALM_wt_ Z score	−1.81*** (−1.93, −1.69)	−0.95*** (−1.34, −0.55)	−0.47** (−0.74, −0.19)	−2.69*** (−3.28, −2.10)	1.31*** (1.05, 1.57)	−13.89*** (−17.49, −10.48)	−0.30*** (−0.35, −0.26)	1.92*** (1.65, 2.22)
ALM_bmi_ Z score	−0.06 (−0.19, 0.06)	−0.85*** (−1.22, −0.47)	−0.05 (−0.31, 0.21)	−1.63*** (−2.19, −1.07)	0.56*** (0.31, 0.81)	−11.87*** (−15.09, −8.65)	−0.15*** (−0.19, −0.11)	1.31*** (1.13, 1.52)
ALM_trunkfat_ Z score	−2.64*** (−2.77, −2.51)	−1.06*** (−1.49, −0.62)	-0.67*** (−0.98, −0.37)	−2.75*** (−3.40, −2.10)	1.69*** (1.40, 1.98)	−19.98*** (−23.74, −16.21)	−0.38*** (−0.43, −0.33)	2.04*** (1.76, 2.36)
Female								
ALMI Z score	0.78*** (0.64, 0.91)	0.27 (−0.12, 0.68)	−0.21 (−0.47, 0.05)	−0.28 (−0.79, 0.24)	−0.71*** (−0.98, −0.43)	−2.09* (–4.03, −0.17)	0.02 (−0.04, 0.08)	1.02 (0.84, 1.24)
ALMI_fmi_ Z score	0.04 (−0.05, 0.12)	0.06 (−0.19, 0.31)	−0.16* (−0.33, −0.00)	−0.19 (−0.52, 0.13)	−0.27** (−0.45, −0.10)	−1.73** (−2.95, −0.52)	−0.03 (−0.06, 0.01)	0.99 (0.85, 1.16)
ALM_wt_ Z score	−0.97*** (−1.09, −0.85)	−0.31 (−0.66, 0.04)	−0.18 (−0.40, 0.04)	-0.68** (−1.13, −0.23)	−0.04 (−0.27, 0.20)	−3.72*** (−5.38, −2.06)	−0.11*** (−0.16, −0.06)	1.14 (0.98, 1.33)
ALM_bmi_ Z score	0.27*** (0.15, 0.38)	−0.25 (−0.58, 0.09)	0.12 (−0.10, 0.33)	−0.16 (−0.59, 0.27)	−0.13 (−0.36, 0.09)	−3.24*** (−4.84, −1.63)	−0.06* (−0.11, −0.01)	0.97 (0.83, 1.12)
ALM_trunkfat_ Z score	−2.07*** (−2.19, −1.94)	−0.86*** (−1.24, −0.47)	−0.44** (−0.69, −0.19)	−1.82*** (−2.32, −1.32)	0.89*** (0.63, 1.15)	−11.26*** (−13.09, −9.40)	−0.25*** (−0.30, −0.19)	1.45*** (1.25, 1.67)

BMI = body mass index; ALM = appendicular lean mass; ALMI = appendicular lean mass index; FM = fat mass; FMI = fat mass index; ALMI_fmi_ = appendicular lean mass index adjusted for fat mass index; ALM_wt_ = appendicular lean mass to weight; ALM_bmi_ = appendicular lean mass to body mass index; ALM_trunkfat_ = appendicular lean mass to truncal fat; SBP = systolic blood pressure; DBP = diastolic blood pressure; HDL = high-density lipoprotein cholesterol; TG = triglyceride; HOMA-IR = homeostatic model assessment for insulin resistance.

*p value < 0.05, **p value < 0.01, ***p value < 0.001.

When analysed with multivariable logistic model, each index was strongly associated with metabolic syndrome in male, but again the ALM_trunkfat_ was the only index associated with metabolic syndrome in female (odds ratio = 1.45, 95% confidence interval [CI]: 1.25, 1.67).

### Comparison of receiver operating characteristic curves in metabolic syndrome among different indices of low muscle mass

The receiver operating characteristic (ROC) curves of different indices of low muscle mass in predicting metabolic syndrome are shown in Fig. 2 in male and Fig. 3 in female. The area under the receiver operating characteristics (AUCs) of ALMI, ALMI_fmi_, ALM_bmi_ and ALM_wt_ showed moderately good discrimination for metabolic syndrome. The AUCs of ALM_trunkfat_ were 0.74 (95% CI: 0.73, 0.75) in male and 0.69 (95% CI: 0.68, 0.70) in female, indicating that ALM_trunkfat_ was the best discrimination index for metabolic syndrome.

The ROC contrast estimation showed that when compared with ALMI, the ALM_wt_ (difference 0.051, 95% CI: 0.029, 0.073) and ALM_trunkfat_ (difference 0.091, 95% CI: 0.071, 0.110) were better index for metabolic syndrome in male. In female, the same estimation revealed that ALMI_fmi_ (difference −0.065, 95% CI: −0.071, −0.060) and ALM_wt_ (difference −0.061, 95% CI: −0.081, −0.060) were not better than ALMI index, but ALM_trunkfat_ performed better (difference 0.040, 95% CI: 0.022, 0.059) than ALMI.

Table 4 shows the different AUCs of ALM_trunkfat_ in various age, BMI and sex groups. When a person’s BMI is normal weight (BMI< 23) or overweight (BMI between 23 and 25), the ALM_trunkfat_ index performed significantly better in predicting metabolic syndrome. However, when a person’s BMI is more than 30, the ALMI_fmi_ index in male or the ALMI index in female modestly worked, but performed better than the ALM_trunkfat_ index in predicting metabolic syndrome. Based on maximised Youden’s index, cut-off values of 2.60 in male (sensitivity 76.2% and specificity 62.1%) and 1.44 in female (sensitivity 76.6% and specificity 67.0%), which were the optimal cut-off values of the ALM_trunkfat_ index in predicting metabolic syndrome, were obtained^20^.

**Table 4:** Best ROC models of decreased muscle mass index in metabolic syndrome across sex, age and body mass groups.

Sex	Age group	BMI <23		23≤ BMI< 25		25≤ BMI <30		BMI ≥30	
		ROC model	Area (95% CI)	ROC model	Area (95% CI)	ROC model	Area (95% CI)	ROC model	Area (95% CI)
Male	20–34	ALM_trunkfat_	0.71 (0.52,0.89)	ALM_trunkfat_	0.78 (0.68, 0.88)	ALM_trunkfat_	0.58 (0.51, 0.66)	ALMI_fmi_	0.64 (0.51, 0.77)
	35–49	ALM_trunkfat_	0.74 (0.68, 0.80)	ALM_trunkfat_	0.62 (0.56, 0.68)	ALM_trunkfat_	0.63 (0.59, 0.66)	ALMI_fmi_	0.64 (0.51, 0.77)
	50–64	ALM_trunkfat_	0.70 (0.64, 0.75)	ALM_trunkfat_	0.65 (0.60, 0.70)	ALM_trunkfat_	0.62 (0.58, 0.66)	ALM_trunkfat_	0.59 (0.24, 0.94)
	≥65	ALM_trunkfat_	0.72 (0.67, 0.77)	ALM_trunkfat_	0.63 (0.57, 0.68)	ALM_trunkfat_	0.67 (0.61, 0.72)	ALM_trunkfat_	0.64 (0.21, 1)
Female	20–34	ALM_trunkfat_	0.63 (0.27, 0.99)	ALM_trunkfat_	0.72 (0.55, 0.89)	ALM_trunkfat_	0.65 (0.56, 0.75)	ALMI_fmi_	0.62 (0.45, 0.79)
	35–49	ALM_trunkfat_	0.75 (0.67, 0.84)	ALM_bmi_	0.59 (0.51, 0.67)	ALMI	0.62 (0.57, 0.67)	ALMI	0.66 (0.56, 0.76)
	50–64	ALM_trunkfat_	0.61 (0.56, 0.67)	ALM_trunkfat_	0.58 (0.53, 0.62)	ALM_trunkfat_	0.58 (0.54, 0.62)	ALMI	0.52 (0.40, 0.65)
	≥65	ALM_trunkfat_	0.68 (0.64, 0.72)	ALM_trunkfat_	0.55 (0.50, 0.60)	ALM_trunkfat_	0.61 (0.57, 0.66)	ALMI	0.60 (0.43, 0.77)

BMI = body mass index; ALM = appendicular lean mass; ALMI = appendicular lean mass index; FM = fat mass; FMI = fat mass index; ALMI_fmi_ = appendicular lean mass index adjusted for fat mass index; ALM_wt_ = appendicular lean mass to weight; ALM_bmi_ = appendicular lean mass to body mass index; ALM_trunkfat_ = appendicular lean mass to truncal fat.

## Discussion

In this study, we proved a clinically meaningful index of low muscle mass associated with cardiometabolic risk factors considering age, sex and BMI categories among a Korean representative population.

Increased central adiposity has been well established for adverse cardiometabolic outcomes^21,22^, but the impact of low muscle mass has been recently investigated^12,23^. Several studies regarding low muscle mass and cardiometabolic syndrome have been published. However, they sometimes show conflicting results depending on the index used for muscle mass depletion, in addition to different age and sex^13,24–27^. Recently, a study compared three different indices of low muscle mass associated with cardiometabolic risks among a Korean population using the young reference group. The results showed that ASM/BMI-defined index seems to have a closer relationship with cardiometabolic risks^28^. However, this index did not show a strong association with metabolic syndrome in a Korean population compared with a Caucasian population^29^.

Asians are particularly known to be vulnerable to increased cardiometabolic risks even at the lower BMI^30^. Low muscle mass and decreased mitochondrial function in skeletal muscles have been speculated to contribute to higher risk for insulin resistance and diabetes in Asians^31^. Assessing cardiometabolic risks of Asians just by cut-off of abdominal circumferences or BMI could miss several preventable adverse cardiovascular events or diabetes. In this regard, our study added a significant index of low muscle mass for evaluating cardiometabolic risks, especially at normal BMI.

Wells^32^ first suggested the concept of “metabolic load” of adiposity and “metabolic capacity” of lean mass, and Prado *et al*.^7^ conceptualised the metabolic load-capacity model of sarcopenic obesity instead of absolute muscle mass adjusted by body size. Siervo *et al*.^33^ suggested body composition indices of a load capacity model using reference curves of fat mass to fat-free mass or truncal fat mass to ALM with consideration of sex, age and BMI. They tested the validity of truncal fat mass to ALM in predicting mortality in a population aged between 50 and 70 years old^34^. This concept of metabolic load and capacity model was assessed using the ratio of visceral fat to thigh muscle area by CT scan and proved significant association with metabolic syndrome and incident type 2 diabetes^35,36^. We also confirmed the ALM_trunkfat_ index in relation with cardiometabolic risks across sex, age and most of BMI categories except more than 30.

However, when a subject is obese (BMI >30), we found that absolute muscle mass adjusted for height seems to be more important than metabolic load-capacity model. Since an older population with BMI >30 constitutes a relatively small percentage of population in KNHANES, we could not draw an adequate conclusion with regard to the relevant index of low muscle mass. However, many studies have been performed to evaluate low muscle mass with cardiovascular disease and mortality. Decreased muscle mass with obesity in older adults seems to have more adverse effects on cardiovascular disease and mortality than those with low muscle mass or obesity alone^37^. Since the most important risk factor in cardiometabolic disorder is obesity defined by BMI >30^38^, having low muscle mass defined by ALMI in this population might have added another risk into the already existing cardiometabolic risk profiles.

Considering the effect of complex interplay with fat mass, fat-free mass, influence of sex hormones and ethnic variability on cardiometabolic risks, these factors should be considered altogether in the assessment of clinically meaningful low muscle mass in predicting health status or diseases^8^. In this regard, we adopted the Z-score based on several body composition indices by considering age- and sex-specific influences instead of the simple cut-off value of muscle mass indices compared with the young reference group. We believe that our approach may expand the concept of low muscle mass in relatively young and middle-aged population instead of focusing on older adults.

In this study, we compared muscle mass with fat mass among different indices in the low muscle mass and control groups. The low muscle mass group defined by ALMI showed lower muscle mass with FMI and that by ALM_fmi_ had lower muscle mass with similar FMI, but that defined by ALM_wt_, ALM_bmi_ and ALM_trunkfat_ showed similar muscle mass with higher FMI compared with the control group. Thus, the ALM_fmi_ index seemed to represent a strict definition of low muscle mass at a given FMI, but this index was not associated with metabolic syndrome in our population. This was consistent with another study showing that low muscle mass alone was not a risk factor for metabolic syndrome^26^.

The cross-sectional design of this study limited the causal or temporal relationship between body composition and adverse cardiometabolic risk factors. The KNHANES dataset is instrument specific, and generalisation to different instruments or measurement methods is limited.

We used the Z-score-driven indices of low muscle mass and tested its association with metabolic syndrome using the same dataset, which limited external validation. More rigorous external validation will be needed. Our study also lacked muscle quality measurements, such as strength and gait speed, which are critical for functional assessment in older adults^39^.

Although we suggested crude cut-off values for ALM_trunkfat_ as 2.60 in male and 1.44 in female to predict metabolic syndrome in normal or overweight subjects whose BMI are less than 30, further evaluation and testing age, sex and BMI referenced values for ALM_trunkfat_ will be explored for clinical use.

Despite the several limitations of this study, we compared five recently published indices of low muscle mass in association with cardiometabolic risks in a Korean population. We first adopted the Z-score based on low muscle mass reflecting age- and sex-related body composition changes and proved the usefulness of low muscle mass index in a Korean population.

Our study showed that ALM to truncal fat ratio could be a useful indicator for screening cardiometabolic risk factors, particularly in normal or overweight Asian population who are vulnerable to adverse cardiovascular events even at normal weight^40^. Further evaluation of identifying metabolic risk groups using the low muscle mass index in various ethnic groups is necessary.

## Methods

### Study subjects

**Figure 1:**
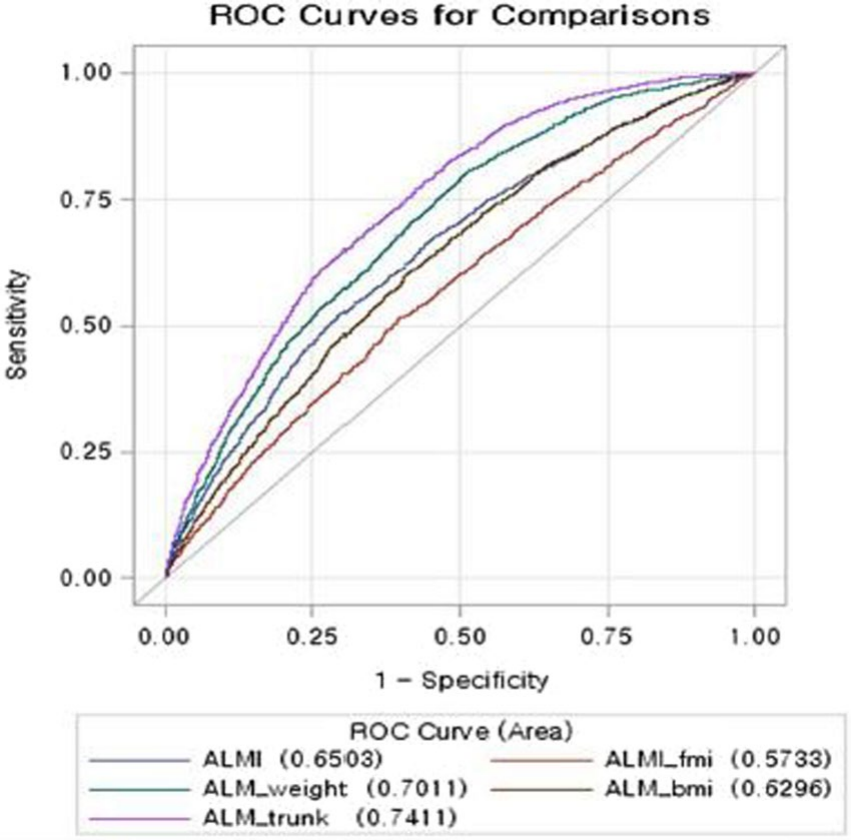
Selection of study participants.

We used data from the fourth and fifth KNHANES conducted from July 2008 to May 2011. These surveys have been conducted periodically since 1998 to assess the health and nutritional status in a representative population of Korea. This was a cross-sectional study conducted by the Korean Ministry of Health and Welfare and the Korea Centers for Disease Control and Prevention. A complex, multistage, probability rolling survey sampling method was used to enrol participants in KNHANES based on geographic area, sex and age group using household registries. There were 19,269 subjects aged between 20 and 100, and the total and regional lean mass with fat mass were measured by DXA (Lunar Corp., Madison, WI, USA). Patients currently receiving cancer treatments or who reported recent weight change more than 10 kg for the last 1 year were excluded. A total of 17,870 subjects (7,667 men, 10,203 women) were finally included in the study population and study flow is illustrated in Fig. 1. All participants in this survey provided written informed consent, and the institutional review board of the Korea Centers for Disease Control and Prevention approved the study protocol. All research activities in this study were conducted in accordance with the ethical principles of the Declaration of Helsinki.

**Figure 2:**
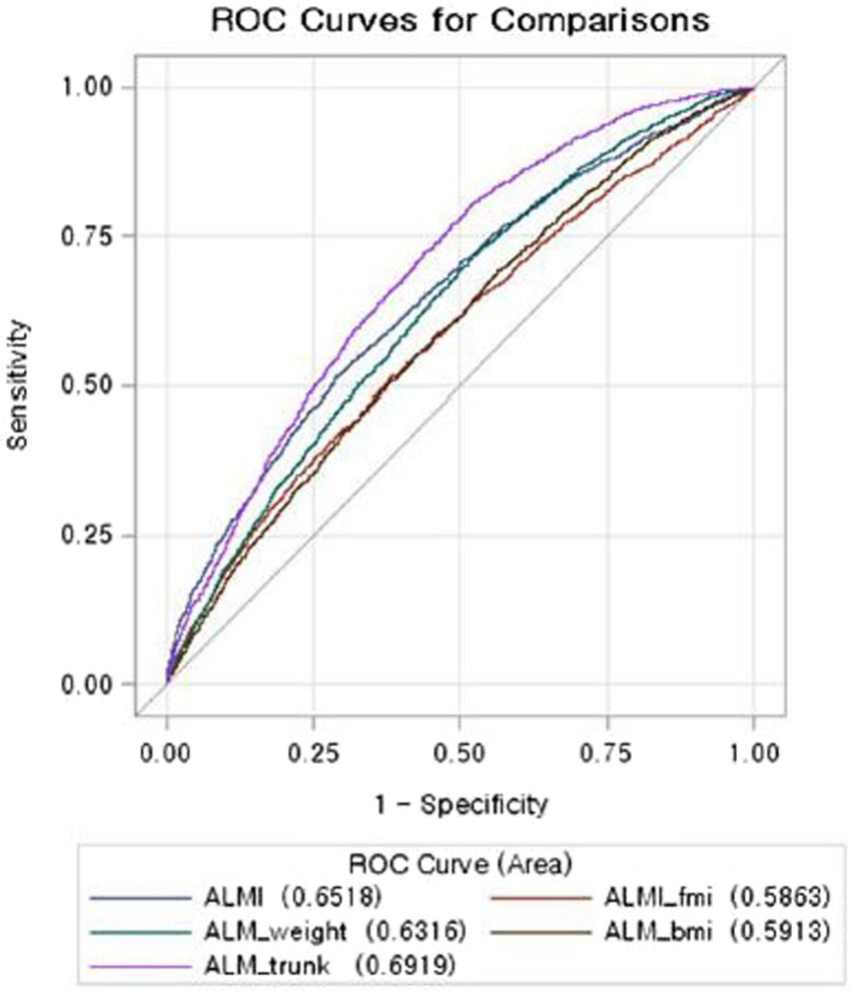
Comparison of ROCs in metabolic syndrome among different low muscle mass indices in male.

**Figure 3:**
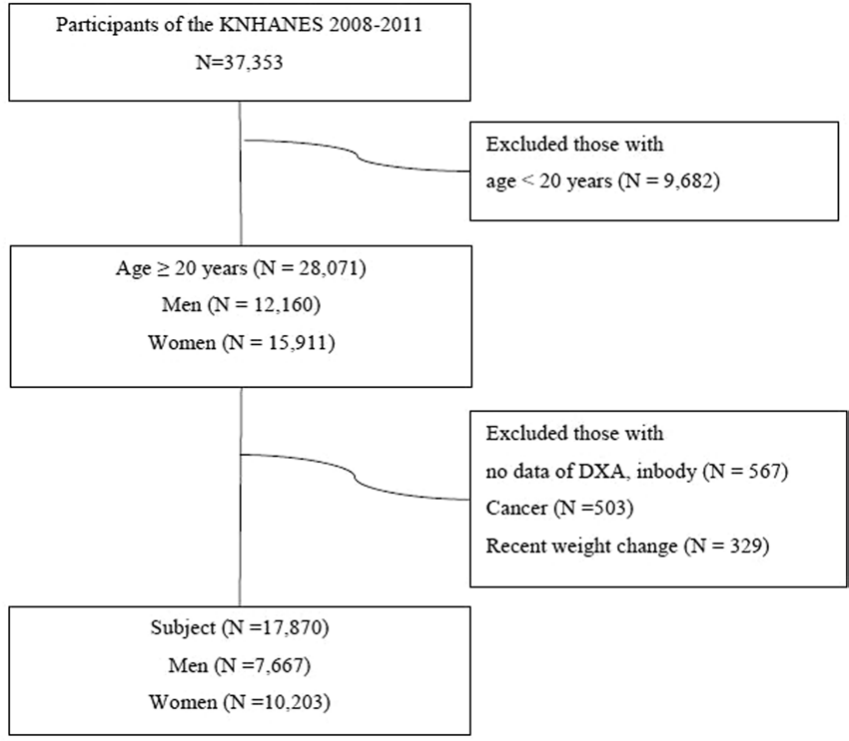
Comparison of ROCs in metabolic syndrome among different low muscle mass indices in female.

### Indices of muscle mass, low muscle mass and low muscle mass for age

Body composition variables included fat mass and lean soft mass measured by DXA.

Five different definitions for muscle mass measurements with low muscle mass were used using DXA. The first index was ALM adjusted by height (ALM/height^2^, ALMI). Since normal lean mass can be determined by age, sex, fat mass and body region, novel definition of ALMI adjusted for FMI (fat mass/height^2^) has been developed and proved its validity in predicting functional impairments among patients with rheumatoid arthritis or chronic kidney disease^41–43^. Thus, sex- and age-specific curves for ALMI and FMI were used using KNHANES data with the lamda, mu, sigma (LMS) method, which is the standard method for expressing body composition as a standardised deviation score^44,45^.

As a second index, we calculated ALMI_FMI_ Z-score and T-score using equations previously developed with NHANES data^41^. Data from KNHANES were used to regress ALMI Z or T score on FMI Z or T score. While the mean beta coefficient of ALMI on the FMI Z score was approximately 0.6 and the addition of FMI^2^ term improved the model fit according to the report using US NHANES, the mean beta coefficient was approximately 0.33 in a Korean population. Since the ALMI Z and FMI Z scores were non-linear, the addition of FMI^2^ term with FMI Z score*ALMI Z score better improved the model fit than the FMI^2^ alone in our model. Thus, the predicted ALMI Z score relative to the FMI Z score using age and sex was as follows:

$${\rm{Predicted}}\,{\rm{ALMI}}\,{\rm{Z}}\,{\rm{score}}={\rm{\beta }}1\,({\rm{FMI}}\,{\rm{Z}}\,{\rm{score}})+{\rm{\beta }}2\,{({\rm{FMI}}{\rm{Z}}{\rm{score}})}^{{\rm{2}}}+{\rm{\beta }}3\,({\rm{FMI}}\,{\rm{Z}}\,{\rm{score}}\ast {\rm{ALMI}}\,{\rm{Z}}\,{\rm{score}})+{\rm{constant}}$$

The third index was the ratio of ALM_wt_. We calculated the ALM_wt_ Z score and T score using the LMS method. The ALM_wt_ shows better correlation with metabolic syndrome than with ALMI in a Korean population^19,46^.

The fourth index was ALM_bmi_, which was developed and tested its validity by the Foundation for the National Institutes of Health Sarcopenia Project^47^. ALM_bmi_ not only shows better relationship with mobility impairment and mortality among older adults^47^ but also with cardiometabolic components compared with ALMI or thigh muscle cross-sectional area adjusted with weight^28^.

The fifth index was the ratio of fat to muscle, which accounted for age, sex and BMI^33^. In particular, the ratio of truncal fat to ALM proved to be a useful diagnostic approach in overall mortality^34^. Several studies found that skeletal muscle mass to visceral fat ratio is associated with metabolic syndrome, nonalcoholic steatohepatitis and arterial stiffness^48–50^. We adopted this index as ALM_trunkfat_ because we focused on assessing and comparing indices of low muscle mass relative to height, weight, BMI, FMI or truncal fat.

Instead of previously used cut-off values compared with young reference group, we used normalised Z score for each index generated by the LMS method from age- and sex-specific original reference curves. The LMS method normalises the data after Box–Cox power transformation to yield a corresponding Z score using smoothed L (skewness), M (median) and S (coefficient of variation). This method addresses skewness, non-linearity and heteroskedasticity, as well as accounts for the effects of age, sex and ethnicity^51^. Measurements of ALMI, ALMI_fmi_, ALM_wt_, ALM_bmi_ and ALM_trunkfat_ were converted to sex- and age-specific Z and T scores based on the LMS values in a 25-year old using the original data as well as published KNHANES LMS data^52^. It should be noted that the aim of the study was to evaluate the most valid index among various indices of low muscle mass in assessing cardiometabolic risks across age and sex. T score-defined low muscle mass was too much deviated in older age with the low BMI group. Thus, we adopted the concept of relative low muscle mass using Z-score in this study.

### Measurements of cardiometabolic risk factors

During the fourth and fifth KNHANES surveys, participants were asked about medical history and health-related behaviours. The participants also underwent physical examination and biochemical measurements by trained medical staff following standardised procedures.

The subjects were categorised as current smokers, ex-smokers if they reported any history of cigarette smoking or never smokers. Alcohol intake was assessed by asking about the usual frequency and amount of consumption over the past year. It was categorised as high-risk intake if subjects had an average daily consumption of alcohol more than 35 g/day based on the results of alcohol intake and risk of metabolic syndrome^53^. Subjects were classified as regular exercisers if they reported moderate- to high-intensity exercise of 30 min per session on multiple days with a total of 150 min of exercise per week^54^.

Weight and height were measured with light clothes and no shoes. BMI was calculated as weight (kg) divided by height in meters squared. Waist circumference was measured at the midline between the inferior margin of the lowest rib and the iliac crest on a horizontal zone. Blood pressure (BP) was measured using a standard mercury sphygmomanometer (Baumaometer; WA Baum Co., Inc., Copiague, NY, USA). Two systolic and diastolic BP readings were recorded and averaged for analysis.

Blood samples were obtained after an 8-h morning fast. Serum total cholesterol, TG, HDL-cholesterol (HDL-C), low-density lipoprotein cholesterol and fasting blood glucose (FBG) were measured with a Hitachi Automatic Analyzer 7600 (Hitachi, Tokyo, Japan) using enzymatic methods. Insulin level was measured by immunoradiometric assay (Biosource, Belgium) with a gamma counter (1470 Wizard; Perkin Elmer, Turku, Finland). The HOMA-IR^55^ was calculated using the following formula: FBG (mg/dL) × fasting insulin (µU/mL)/405.

Metabolic syndrome was defined using the National Cholesterol Education program Adult Treatment Panel III criteria^56^ and adopted the criteria of abdominal obesity by the Korean Society for the Study of Obesity^57^. Subjects were categorised as having metabolic syndrome if they have three or more of the following parameters: (1) waist circumference ≥90 cm for men and ≥85 cm for women, (2) TG level ≥150 mg/dL, (3) FBG ≥100 mg/dL or use of anti-diabetic medication, (4) systolic BP ≥130 mmHg or diastolic BP ≥85 mmHg or use of antihypertensive medication and (5) HDL-C level <40 mg/dL for men and <50 mg/dL for women.

### Statistical analysis

Baseline characteristics of participants were expressed as means and standard errors for continuous variables or as percentages in categorical variables by using the survey means or survey frequency procedure in SAS version 9.3 program (SAS Institute, Cary, NC, USA). A total of 17,890 study participants were stratified by sex, age group and BMI. Age was classified as 20 to 34 years, 35 to 49 years, 50 to 64 years and more than 65 years. BMI categories were classified as normal weight (BMI< 23), overweight (23≤ BMI< 25), obese (25≤ BMI< 30) and morbid obese (BMI ≥30) according to the criteria proposed by the Regional Office for Western Pacific Region of WHO^58^.

The LMS method was applied to generate age- and sex-specific reference curves among different indices of low muscle mass using LMS chartmaker Pro version 2.5.4^59^. Sex- and age-specific reference curves for ALMI, ALMI_fmi,_ ALM_wt_, ALM_bmi_ and ALM_trunkfat_ were applied to yield a corresponding Z score as the following equation:

$${\rm{Z}}=[{({\rm{measure}}/{\rm{M}})}^{{\rm{L}}}-1]/({\rm{L}}\ast {\rm{S}})$$

Z: standardized z score for each index, measure: low muscle mass index, L: lambda, M: mu, S: sigma.

Each index derived from the LMS method was evaluated with cardiometabolic components such as waist circumference, FBG, systolic BP, diastolic BP, TG, HDL-C or HOMA-IR using linear regression methods adjusted with age, sex and BMI categories. We also explored the association of different definitions of low muscle mass with metabolic syndrome using logistic regression methods and calculated odds ratio.

Finally, five indices of low muscle mass were compared in relation to metabolic syndrome using the AUC curves in each sex, age and BMI categories and in general. Two-sided p values lower than 0.05 were considered significant.
