# Author Correction: Comparisons of different indices of low muscle mass in relationship with cardiometabolic disorder

**DOI:** 10.1038/s41598-020-57828-8

**Published:** 2020-01-15

**Authors:** Ju Young Kim, Sohee Oh, Hwa Yeon Park, Ji Hye Jun, Hwa Jung Kim

**Affiliations:** 1 Department of Family Medicine, Seoul National University Bundang Hospital, Seoul National University College of Medicine, Seongnam, Korea; 2 Department of Biostatistics, Seoul Metropolitan Government Seoul National University Boramae Medical Center, Seoul, Korea; 3 Department of Family Medicine, Seoul National University Bundang Hospital, Seongnam, Korea; 4 Deparment of Family Medicine, Chamjoeun Hospital, Gwangju, Korea; 5 Department of Preventive Medicine, University of Ulsan College of Medicine, Seoul, Korea

Correction to: *Scientific Reports* 10.1038/s41598-018-37347-3, published online 24 January 2019

In this Article, the legend of Figure 1 is incorrect:

“Selection of study participants”

should read:

“Comparison of ROCs in metabolic syndrome among different low muscle mass indices in male”

Additionally, the legend of Figure 2 is incorrect:

“Comparison of ROCs in metabolic syndrome among different low muscle mass indices in male”

should read:

“Comparison of ROCs in metabolic syndrome among different low muscle mass indices in female”

In addition, the legend of Figure 3 is incorrect:

“Comparison of ROCs in metabolic syndrome among different low muscle mass indices in female”

should read:

“Selection of study participants”

